# Impaired Blastocyst Formation in *Lnx2*-Knockdown Mouse Embryos

**DOI:** 10.3390/ijms24021385

**Published:** 2023-01-10

**Authors:** Seung-Jae Lee, Jaehwan Kim, Gwidong Han, Seung-Pyo Hong, Dayeon Kim, Chunghee Cho

**Affiliations:** 1 School of Life Sciences, Gwangju Institute of Science and Technology, Gwangju 61005, Republic of Korea; 2 Developmental Epigenetics Laboratory, Department of Animal Science, Michigan State University, East Lansing, MI 48824, USA

**Keywords:** *Lnx2*, Notch signaling, preimplantation embryo, blastocyst, inner cell mass

## Abstract

Ligand of Numb-protein X 2 (LNX2) is an E3 ubiquitin ligase that is known to regulate Notch signaling by participating in NUMB protein degradation. Notch signaling is important for differentiation and proliferation in mammals, and plays a significant role in blastocyst formation during early embryonic development. In this study, we investigated *Lnx2* in mouse preimplantation embryos. Expression analysis showed that *Lnx2* is expressed in oocytes and preimplantation embryos. *Lnx2*-knockdown embryos normally progress to the morula stage, but the majority of them do not develop into normal blastocysts. Transcript analysis revealed that the expression levels of genes critical for cell lineage specification, including octamer-binding transcription factor 4 (*Oct4*), are increased in *Lnx2* knockdown embryos. Furthermore, the expression levels of Notch and Hippo signaling-related genes are also increased by *Lnx2* knockdown. Collectively, our results show that *Lnx2* is important for blastocyst formation in mice, suggest that this may act via lineage specification of inner cell mass, and further show that *Lnx2* may be involved in transcriptionally regulating various genes implicated in early embryonic development.

## 1. Introduction

A preimplantation embryo is a pluripotent cell capable of developing into various cell and tissue types through cell division and differentiation. During early mammalian embryonic development, a series of molecular and cellular events occur [1]. The embryos rapidly divide by cleavage without cell growth and thereby develop from 2- to 16- cell stages. Thereafter, blastomeres maximally attach to one another by compaction and a fluid-filled space is generated inside the embryo by cavitation. This forms a blastocyst that is ready for implantation. These events are tightly regulated by the specific modulation of gene expression. Immediately after fertilization, the embryo first develops using only maternal mRNAs and proteins. At the 2-cell stage in mice, transcription is initiated by zygotic gene activation (ZGA) and maternal mRNAs and proteins are degraded gradually thereafter. At the blastocyst stage, embryonic cells undergo differentiation and separate into the inner cell mass (ICM) and trophectoderm (TE) [2,3]. This cell lineage specification is regulated by various cell signaling pathways. The Notch signaling pathway is a key regulator of cell fate determination and differentiation during early embryonic development [4,5]. The Hippo signaling pathway is an important regulator of cell growth, cell polarity and development [4,6]. The Notch and Hippo pathways cooperatively regulate the binary cell fate determination between ICM and TE.

Members of the LNX (Ligand of Numb-protein X) protein family comprise a RING (Really Interesting New Gene) domain and PDZ (PSD95, DLGA, ZO-1) domains. LNX1 and LNX2 share the same domain architecture and similar motifs [7]. The RING domain confers ligase function by binding ubiquitination enzymes and their substrates, and many RING domain-containing proteins induce degradation of target proteins through the ubiquitination pathway [8]. PDZ domains mediate protein-protein interaction through binding to carboxyl terminal sequence motifs of target proteins [9]. The mouse embryo single cell RNA-seq dataset (GSE128691) [10] of the Gene Expression Omnibus (GEO) database at the National Center for Biotechnology Information (NCBI) indicates that, during early embryonic development, *Lnx1* is rarely expressed but *Lnx2* shows relatively high-level expression. Thus, *Lnx2* may function during early embryonic development. LNX2 is an E3 ubiquitin ligase that is known to interact with the NUMB protein and promote its degradation [11]. The NUMB protein inhibits Notch signaling by degrading Notch1 through the ubiquitination pathway [12]. Some recent studies showed that *Lnx2* knockdown induces accumulation of the NUMB protein and thereby inhibits Notch signaling [13,14], whereas others found that overexpression or knockdown of *Lnx2* altered NUMB protein expression but did not affect the Notch signaling output [15,16]. Therefore, the effect of *Lnx2* on Notch signaling is unclear.

In this study, we explored the expression and function of *Lnx2* in preimplantation mouse embryos. We found that *Lnx2* is expressed in oocytes and preimplantation embryos, and knockdown analysis revealed that *Lnx2* is important for blastocyst formation. Interestingly, *Lnx2*-knockdown embryos exhibited increases in the transcription levels of various embryonic genes, including inner cell mass-specific genes such as octamer-binding transcription factor 4 (*Oct4*), as well as Notch target genes and a Hippo signaling pathway-related gene.

## 2. Results

### 2.1. Lnx2 Expression in Various Tissues and Early Embryos

**Figure 1:**
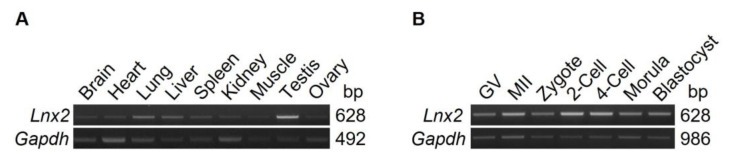
*Lnx2* was ubiquitously expressed in various tissues and preimplantation embryos. (**A**) All tissues, except ovary, were obtained from adult male mice and cDNA was synthesized. Ovary and (**B**) early embryos, including oocytes, were obtained from adult female mice and cDNA was synthesized. Germinal vesicle (GV) and metaphase II (MII) oocytes were obtained at 48 h post PMSG injection, while zygotes and 2-cell, 4-cell, morula, and blastocyst-stage embryos were obtained at 48 h, 60 h, 90 h, 120 h post hCG injection, respectively. *Lnx2* expression was normalized to that of *Gapdh*.

*Lnx2* has been reported to be expressed in various tissues including the brain, heart and testis [7,17]. However, its transcription in ovary and early embryos is unclear. To investigate the expression pattern of *Lnx2*, we performed reverse transcription-polymerase chain reaction (RT-PCR) in various adult mouse tissues and early embryos. *Lnx2* transcripts of the expected size were detected weakly but apparently in ovary and other tissues (Figure 1A and Figure S1A). *Lnx2* transcription was also observed during oocyte maturation and preimplantation embryogenesis (Figure 1B). *Lnx2* transcripts appeared to be slightly more abundant in 2- and 4-cell embryos, in which cell division occurs and zygotic gene activation (ZGA) is initiated, than in the other stages (Figure S1B). These observations suggest that *Lnx2* may be involved in embryonic development.

### 2.2. Lnx2 Knockdown Impairs Blastocyst Formation

**Figure 2:**
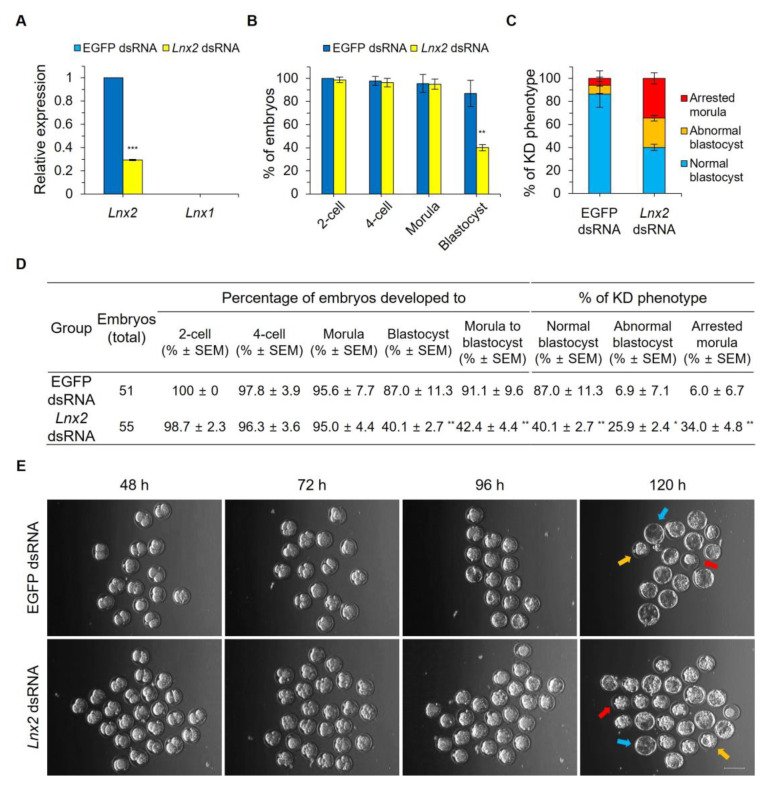
*Lnx2* dsRNA-injected embryos failed to develop into the blastocysts. Zygotes (24 h post hCG injection) were injected with 1 mg/mL of *Lnx2* dsRNA or 1 mg/mL of EGFP dsRNA (injection control). (**A**) cDNA was synthesized from morula stage embryos at 90 h post hCG injection. Values represent means ± SD *** *p* < 0.001 (experiments were repeated three times, Student’s *t* test). Expression levels of *Lnx2* and *Lnx1* were normalized to that of *Gapdh*. (**B**) The development of embryos was observed from 2-cell to blastocyst stages (up to 120 h post hCG injection). EGFP dsRNA-injected embryos (*n* = 51) and *Lnx2*-knockdown embryos (*n* = 55) were used for knockdown analysis. Data are presented as mean ± SEM; ** *p* < 0.01 (three biological replicates, Student’s *t* test). (**C**) Control and *Lnx2*-knockdown embryos were separated based on morphology at the blastocyst stage. The error bars represent SEM. (**D**) Table showing the data graphed above. Data are presented as mean ± SEM. Two-tailed Student’s *t* tests were used for statistical analysis (** *p* < 0.01, * *p* < 0.05). (**E**) Micrographs showing each stage in the development of control and *Lnx2*-knockdown embryos observed at 48 h, 72 h, 96 h and 120 h post hCG injection. Scale bar, 100 μm. Blue arrow indicates a normal blastocyst, orange arrow indicates an abnormal blastocyst with a small cavity, and red arrow indicates an arrested morula.

To determine the function of *Lnx2* in preimplantation embryos, we performed knockdown analysis by microinjecting *Lnx2* double-strand RNA (dsRNA) into mouse zygotes. Real-time quantitative RT-PCR (qRT-PCR) analysis of knockdown embryos showed that the expression of *Lnx2* was reduced by 71% at the morula stage, compared to control embryos injected with enhanced green fluorescent protein (EGFP) dsRNA (*p* < 0.001) (Figure 2A). This indicates that *Lnx2* knockdown persists until the later stage of embryogenesis. We examined whether *Lnx1* was affected by *Lnx2* knockdown and found that *Lnx1* was not detected in control or knockdown embryos. This suggests that *Lnx2* is not compensated for by *Lnx1* in the knockdown embryos. We observed the development of control and *Lnx2* knockdown embryos and found that both groups developed at a similar rate into the 2-cell, 4-cell, and morula stages. However, while 87.0% of embryos injected with EGFP dsRNA developed into blastocysts, only 40.1% of embryos injected with *Lnx2* dsRNA developed into blastocysts (*p* < 0.01). More than half of *Lnx2*-knockdown embryos failed to progress to the blastocyst stage (Figure 2B). At 120 h post hCG injection (96 h after microinjection), when most of the control embryos had reached the blastocyst stage (Figure 2C–E, blue arrow), 25.9% of *Lnx2* dsRNA-injected embryos showed defective blastocyst formation with small cavities (Figure 2C–E, orange arrow). An additional 34.0% failed to develop beyond the morula stage or collapsed (Figure 2C–E, red arrow).

**Figure 3:**
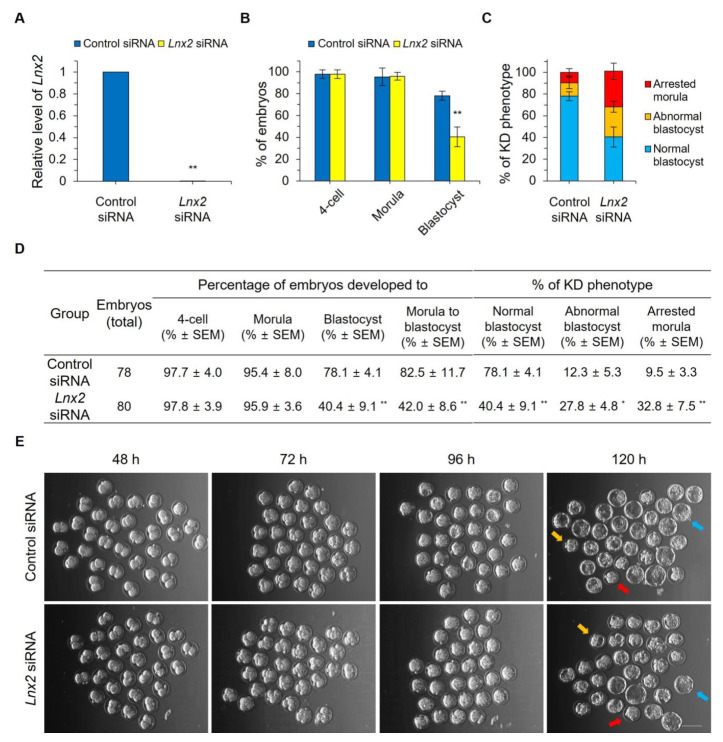
*Lnx2* siRNA-injected embryos failed to develop into the blastocysts. Two-cell embryos (48 h post hCG injection) were injected with 20 μM of *Lnx2* siRNA mixture or control siRNA (injection control). (**A**) cDNA was synthesized from morulae at 90 h post hCG injection. Values represent means ± SD; ** *p* < 0.01 (experiments were repeated three times, Student’s *t* test). Expression levels of *Lnx2* was normalized to that of *Gapdh*. (**B**) The development of embryos was observed from the 4-cell to blastocyst stages (up to 120 h post hCG injection). Control siRNA-injected embryos (*n* = 78) and *Lnx2* siRNA-injected embryos (*n* = 80) were used for knockdown analysis. Data are presented as mean ± SEM; ** *p* < 0.01 (three biological replicates, Student’s *t* test). (**C**) Control and *Lnx2*-knockdown embryos were separated based on morphology at the blastocyst stage. The error bars represent SEM. (**D**) The table shows the data graphed above. Data are presented as mean ± SEM. Two-tailed Student’s *t* tests were used for statistical analysis (** *p* < 0.01, * *p* < 0.05). (**E**) Micrographs for control and *Lnx2*-knockdown embryos of each stage, observed at 48 h, 72 h, 96 h, and 120 h post hCG injection. Scale bar, 100 μm. Blue arrow indicates a normal blastocyst, orange arrow indicates an abnormal blastocyst with a small cavity and red arrow indicates an arrested morula.

In general, dsRNA is cleaved into five or more small interfering RNA (siRNAs) via Dicer [18]. Thus, the dsRNA could have off-target effects. To examine whether the knockdown phenotype was attributable only to the reduction of *Lnx2*, we performed knockdown analysis using siRNA. Because the knockdown effect of siRNA may not last as long as that dsRNA, injection into 2-cell stage embryos, rather than zygotic injection, is recommended [19,20]. Indeed, when we injected *Lnx2* siRNA mixture into zygotes, the transcript expression level of *Lnx2* was significantly decreased at the 2-cell stage but restored at the morula stage (Figure S2). This suggests that the knockdown effect of siRNA did not persist until the morula stage. When the *Lnx2* siRNA mixture was injected into each blastomere at the 2-cell stage, the *Lnx2* transcript level was almost decreased at the morula stage, compared to control embryos (Figure 3A). We found that the siRNA-mediated knockdown phenotype was highly similar to that obtained using dsRNA. In control groups, 78.1% of embryos progressed to the blastocyst stage. In contrast, only 40.4% of embryos injected with *Lnx2* siRNA showed normal blastocyst formation (Figure 3B). Consistent with the results obtained using the *Lnx2* dsRNA, the numbers of abnormal blastocysts and arrested morulae were significantly increased in *Lnx2* siRNA-injected embryos (Figure 3C–E). Although compaction was observed at the morula stage in *Lnx2* siRNA-injected embryos, it appeared that blastocoel formation and separation into ICM and TE did not occur properly in these embryos. Collectively, these results suggest that *Lnx2* is critical for blastocyst formation during preimplantation embryogenesis in mice.

### 2.3. Expression of ICM-Related Gene in Lnx2 Knockdown Embryos

**Figure 4:**
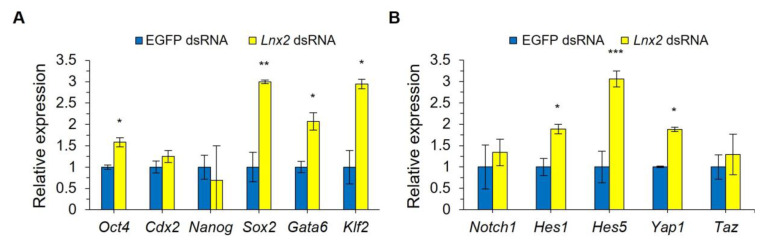
Expression of genes important for early embryonic development in *Lnx2*-knockdown embryos. Zygotes (24 h post hCG injection) were injected with 1 mg/mL of EGFP dsRNA or *Lnx2* dsRNA and cDNA was synthesized from embryos at 90 h post-hCG injection. (**A**) Expression levels of the ICM markers, *Oct4* and *Cdx2*, (**B**) Notch and Hippo pathway genes normalized to that of *Gapdh*. Data are presented as mean ± SEM; *** *p* < 0.001, ** *p* < 0.01, * *p* < 0.05 (three biological replicates, Student’s *t* test).

OCT4 and caudal-type homeobox 2 (Cdx2) proteins are specifically expressed in inner and outer blastomeres, respectively, and act as important regulators in the development of ICM and TE during blastocyst formation. Here, we used real-time qRT-PCR measurement of mRNA to examine whether *Lnx2* knockdown affects the expression levels of *Oct4* and *Cdx2* in late morula stage (90 h post hCG injection, i.e., 66 h after microinjection, representing final stage prior to formation of a fluid filled cavity). We found that *Oct4* transcription was significantly increased in *Lnx2*-knockdown embryos (Figure 4A), whereas *Cdx2* expression was unchanged. Consistently, our immunostaining analysis showed enhanced OCT4 signals in *Lnx2* knockdown embryos at 90 h post hCG injection (i.e., 66 h after microinjection) (Figure S3). To investigate whether ICM was further affected by *Lnx2* knockdown, we examined the expression of *Oct4*-regulated ICM markers, such as Nanog homeobox (*Nanog*), SRY (sex-determining region Y)-box2 (*Sox2*), GATA binding protein 6 (*Gata6*), and Kruppel-like factor2 (*Klf2*). In *Lnx2*-knockdown embryos, the expression level of these genes encoding ICM markers, except *Nanog*, were significantly increased in late morula stage (90 h post hCG injection, i.e., 66 h after microinjection) (Figure 4A). These results suggest that the abnormal development of *Lnx2*-knockdown embryos may be due to disruption of the balance between *Oct4* and *Cdx2*. Therefore, *Lnx2* may be involved in ICM specification through regulation of ICM-related gene expression.

### 2.4. Lnx2 Knockdown Alters the Expression Levels of Genes Involved in Cell Signaling Pathways

LNX2 is an E3 ubiquitin ligase reported to regulate Notch signaling by promoting NUMB protein degradation [11]. To investigate whether LNX2 regulates Notch signaling, we examined the expression of Notch target genes, such as those encoding Hes family bHLH transcription factors 1 and 5 (*Hes1* and *Hes5*), through qRT-PCR. The expression levels of *Hes1* and *Hes5*, were significantly increased in *Lnx2*-knockdown embryos compared to control embryos, but there was no change in the transcript level of *Notch1* (Figure 4B). If *Lnx2* is involved in NUMB degradation, Notch signaling should be inactive in *Lnx2*-knockdown embryos [13]. Since the expression levels of Notch target genes were rather increased by *Lnx2* knockdown, it is likely that *Lnx2* regulates Notch signaling independently of NUMB protein. Finally, we examined the expression of Hippo pathway genes, such as yes-associated protein (*Yap1*) and transcriptional coactivator with PDZ-binding motif (*Taz*), in *Lnx2*-knockdown embryos. The transcript level of *Yap1*, but not *Taz*, was significantly increased by *Lnx2* knockdown (Figure 4B).

Collectively, our results revealed that *Lnx2* knockdown leads to increases in the expression levels of genes known to be critical for embryonic development. Our results may imply that *Lnx2* is largely involved in transcriptional repression during preimplantation embryogenesis, particularly blastocyst formation (see Section 3).

## 3. Discussion

*Lnx2* has been studied in several species and tissues, but not in early mammalian embryos, and the involvement of NUMB in the *Lnx2*-associated regulation of Notch signaling is controversial [21]. Previous reports indicated that *Lnx2* knockdown decreased the expression of the Notch target gene, *Hes1*, in bon-derived macrophages [13] but did not affect the Notch pathway in a pancreatic cancer cell line [15]. Since *Lnx2* appears to have different functions in different cell types, one cannot predict its specific function in early embryogenesis.

In this study, we found for the first time that *Lnx2* is important for early embryonic development. *Lnx1*, which is a LNX gene family member that is closely related to *Lnx2*, did not compensate for *Lnx2* knockdown. Although *Lnx2*-knockdown embryos generally showed normal development up to the morula stage, the majority of embryos did not develop into blastocysts. Some formed small cavities exhibiting incomplete development, while others remained compacted or collapsed at the blastocyst stage. These observations strongly suggest that ICM and TE are not properly segregated in *Lnx2*-knockdown embryos, leading to impaired blastocoel formation. Cell specification for this segregation is mainly regulated by *Oct4* and *Cdx2*, which are critical for ICM formation [3] and TE formation [2], respectively. Specifically, the ICM consists of a mixture of the epiblast (EPI) and primitive endoderm (PE) [22]. In EPI, *Oct4*, *Sox2* [23], *Nanog* [24], and *Klf2* [25] are strongly expressed, whereas PE exhibits abundant expression of *Gata6* [26]. And *Klf2* and *Oct4* activate each other [27,28]. These genes function as ICM markers. Our analysis revealed that transcription of *Oct4* was increased in *Lnx2*-knockdown embryos, compared to controls. The transcript expression levels of other ICM markers (*Sox2*, *Gata6*, *Klf2*) were also increased. These results suggest that *Lnx2* knockdown alters the properties of ICM, resulting in impaired blastocyst formation. One might propose that the altered expression of genes encoding ICM markers in *Lnx2*-knockdown embryos is a result rather than a cause of the abnormal cell allocation and/or segregation seen during blastocyst formation. However, we judge this possibility to be unlikely, given that *Cdx2* expression remained unchanged in *Lnx2* knockdown embryos.

To further investigate the function of *Lnx2*, we examined several signaling pathways. In embryogenesis, Notch signaling is believed by some to be regulated by LNX2 through NUMB. Notch signaling is initiated by the interaction of a ligand with a transmembrane receptor. This leads to the cleavage of the Notch1 intracellular domain (NICD) and its translocation to the nucleus. NICD regulates the transcription of Notch target genes by de-repressing the transcription complex Rbpj (recombination signal binding protein for immunoglobulin kappa J region). In this process, LNX2 regulates NUMB, leading to Notch1 degradation. Thus, we expected that Notch signaling would be activated upon *Lnx2* knockdown. However, we found that the expression levels of the Notch target genes, *Hes1* and *Hes5,* were increased in *Lnx2*-knockdown embryos, suggesting that Notch signaling is upregulated in this case. This implies that the *Lnx2*-mediated regulation of Notch signaling is independent of NUMB in mouse embryos. 

In the nucleus, NICD activates Notch signaling by replacing the repressor complex with an activator complex containing CSL (CBF1, Suppressor of Hairless, Lag-1) and MAM (Mastermind) [29]. In this pathway, LNX2 may affect the repressor complex. In zebrafish, Lnx2b stabilizes the repressor complex formed by transcription factor 3 (TCF3) and histone deacetylase 1 (HDAC1) [30]. TCF3 is a major transcription factor that regulates *Oct4*, *Nanog* and *Sox2* in embryonic stem (ES) cells [31,32], and HDAC1 is a core component of the repressor complex in Notch signaling [33,34]. Lnx-l (Lnx-like) is known to regulate Bozozok, which is a homeodomain-containing transcriptional repressor, in zebrafish embryo [35]. In addition, LNX1, which is similar to LNX2, is known to interact with the transcriptional regulator SKI-interacting protein (SKIP), which constitutes the activator complex in Notch signaling [36]. Some studies have suggested that the LNX family is involved in transcriptional regulation. Therefore, *Lnx2* may play a role in maintaining or activating repressor complex during early embryonic development. 

The Hippo signaling pathway is also important for early embryonic development [4,6]. Inactivation of the Hippo kinase cascade (angiomotin or macrophage-stimulating 1/2) causes dephosphorylation and nuclear translocation of YAP/TAZ. In addition, YAP/TAZ activates target genes by binding to TEA domain transcription factor (TEAD) 1~4. The YAP/TAZ-TEAD complex activates target genes. A recent study revealed that *Oct4* is increased and *Cdx2* is decreased in *Tead4*-knockout embryos [37]. In blastocysts, Notch and YAP/TEAD bind to TE-specific enhancers in the outer cells and activate the TE specification gene, *Cdx2* [4]. However, our study showed that the transcript level of the Hippo pathway gene, *Yap1*, was increased in *Lnx2*-knockdown embryos, whereas that of *Cdx2* was unaltered and that of *Oct4* expression was unexpectedly increased. We do not know for sure how *Lnx2* is related to Hippo and Notch pathway activity and/or whether *Lnx2* could be involved in the general transcriptional repression of embryonic genes through a yet unknown mechanism.

In conclusion, our present study reveals that *Lnx2* is important for early embryonic development, particularly blastocyst formation. To answer whether *Lnx2* is an absolute requirement for blastocyst formation in vivo requires investigation of embryonic development in mice lacking LNX2. *Lnx2* regulates the expression levels of genes encoding ICM markers, including *Oct4*, as well as those of genes involved in the Notch and Hippo signaling pathways. *Lnx2* may directly or indirectly contribute to large-scale transcriptional regulation in mouse embryos. This potential regulatory mechanism involving *Lnx2* deserves future investigation.

## 4. Materials and Methods

### 4.1. Mouse Embryo Collection and Culture

Oocytes and embryos were collected from 8- to 10-week-old B6D2F1 female mice, as described previously [20]. Briefly, female mice were superovulated by injection of 7.5 international units (IU) of pregnant mare serum gonadotropin (PMSG) followed 48 h later by 7.5 IU of human chorionic gonadotropin (hCG). The mice were then mated with B6D2F1 male mice. Fertilized embryos were collected from the oviducts of sacrificed female mice at 18 h post-hCG injection. In addition, germinal vesicle (GV) and metaphase II (MII) oocytes were collected from superovulated female mice at 48 h post-PMSG injection and 72 h post PMSG injection, respectively. Cumulus cells were removed by pipetting the collected embryos in hKSOM (KSOM with HEPES) containing hyaluronidase (0.3 mg/mL). Thereafter, the embryos were cultured in potassium-supplemented simplex optimized medium (KSOM) at 37 °C in a humidified atmosphere of 5% CO_2_ and 5% O_2_ balanced with N2. All animal experiments were performed in accordance with the guidelines of the Animal Care and Use Committee of Gwangju Institute of Science and Technology (GIST-2021-021).

### 4.2. RNA Extraction and RT-PCR

Total RNA was extracted from various tissues (brain, heart, lung, liver, spleen, kidney, muscle, testis) of male mice and ovaries of female mice using TRIzol (Molecular Research Center, Cincinnati, OH, USA) according to the manufacturer’s protocol. cDNA was synthesized using random hexamers, oligo dT, and Omniscript reverse transcriptase (Qiagen, Germantown, MD, USA). For processing of mRNA from embryos, mRNA was isolated from 15–50 preimplantation embryos using a Dynabead RNA Isolation Kit (Thermo Fisher Scientific, Waltham, MA, USA) according to the manufacturer’s protocol. cDNA was synthesized using random hexamers, oligo dT, and a Sensiscript reverse transcriptase (Qiagen), owing to the small amount of RNA in each embryo. RT-PCR was conducted using the primers listed in Table S1, and the results were normalized to the level of *Gapdh*.

### 4.3. Real-Time qRT-PCR

mRNA was isolated from 15–50 preimplantation embryos and reverse-transcribed to cDNA as described above. qRT-PCR was conducted using 10 ul of Sybr Green Taq polymerase mix (Enzynomics, Daejeon, Republic of Korea) and 100–200 ng of template cDNA. qRT-PCR was conducted using the primers listed in Table S1. The 2^−ΔΔCt^ method was used to evaluate relative gene expression levels normalized to *Gapdh* levels. Values are presented as means ± SD. Two-tailed Student’s *t* test was used for statistical analysis and *p* < 0.05 was taken as indicating a significant difference.

### 4.4. Generation of dsRNA and siRNA

dsRNA regions were generated using pBluescript II KS vector, as described previously [20]. dsRNA regions in *Lnx2* and EGFP were generated by PCR using mouse embryo cDNA as the template. Purified sense and antisense strand sequences of dsRNA regions were cloned into the pBlueScript II KS vector, which contains the T7 promoter site. Each strand sequence was PCR amplified with oligonucleotides linked to the T7 promoter region and the M13 universal primer. Amplified strands were purified using a LaboPass PCR Purification Kit (Cosmogenetech, Seoul, Republic of Korea). For each strand, RNA was synthesized using the HiScribe T7 High Yield RNA synthesis Kit (New England Biolabs, Ipswich, MA, USA). For the annealing step, equimolar quantities of sense and antisense RNAs were mixed in annealing buffer (10 mM Tris, pH 7.4, 0.1 mM EDTA), heated for 10 min at 80 °C, and incubated at 37 °C for 3 h. To prevent contamination by single-strand RNA (ssRNA) or proteins, 1 µg/mL RNase A (MilliporeSigma, Burlington, MA, USA) plus 0.5 M NaCl and Proteinase K (for proteins) was applied for 30 min at 37 °C. Annealed dsRNA was extracted with LiCl and ethanol, diluted to a final concentration of 1 mg/mL and kept at −80 °C until use. EGFP dsRNA was used as a injection control dsRNA. siRNAs were synthesized by Bioneer (Daejeon, Republic of Korea). The siRNA sequences were as follows: *Lnx2* siRNA1 5′- GTGACGTGTTGCTGAACAT -3′, *Lnx2* siRNA2 5′- ACTTCCTGCAGGAGAAGGA -3′, and *Lnx2* siRNA3 5′- CCTGGTGGTCTGGTGAATA -3′. Injection control siRNA was also synthesized by Bioneer.

### 4.5. Microinjection

Injection micropipettes were manufactured from borosilicate capillary tubes using a Sutter p-97 glass puller (Sutter Instruments, Novato, CA, USA). The injection holder was handcrafted from borosilicate capillary tube. For microinjection, 1 mg/mL of dsRNA was injected into each zygote (24 h post hCG injection) and 20 μM of siRNA was injected into each blastomere of a 2-cell embryo (48 h post hCG injection) through an IM300 microinjector (Narishige, Amityville, NY, USA) mounted on an inverted laboratory microscope (Leica Microsystems, Buffalo Grove, IL, USA).

### 4.6. Immunofluorescence Staining (IF)

Embryos were fixed using 3.7% formaldehyde (MilliporeSigma, Burlington, MA, USA) in PBS with 0.1% bovine serum albumin (BSA) for 30 min at room temperature. Then, embryos were permeabilized using 0.25% Triton X-100 in PBS with 0.1% BSA for 30 min at room temperature. Embryos were blocked in 5% BSA overnight at 4 °C and incubated with a primary antibody in 3% BSA with 0.1% Triton X-100 overnight at 4 °C. Embryos were transferred to the secondary antibodies in 3% BSA with 0.1% Triton X-100 for 30 min at room temperature. Lastly, embryos were incubated with the Hoechst 33342 (MilliporeSigma, Burlington, MA, USA) in 3% BSA with 0.1% Triton X-100 for 30 min at room temperature. An antibody used for immunostaining was mouse anti-Oct-3/4 antibody (1:100; sc-5279; Santa Cruz Biotechnology, Dallas, TX, USA). The fluorescence intensity in embryo was assessed using Image J. The background signal was used for thresholding.

### 4.7. Statistical Analysis

All experiments were performed at least in triplicate. Data are presented as means ± SD unless otherwise indicated. Two-tailed Student’s *t* test was used for statistical analysis and *p* < 0.05 was taken as indicating a significant difference.

## Data Availability

All data presented in this study are available upon request to the corresponding authors.

## Supplementary Materials

The following supporting information can be downloaded at: https://www.mdpi.com/article/10.3390/ijms24021385/s1.

## Abbreviations

LNX	Ligand of Numb protein X
RING	Really Interesting New Gene
PDZ	PSD95, DLGA, ZO-1
ICM	Inner cell mass
TE	Trophectoderm
EPI	Epiblast
PE	Primitive endoderm
ZGA	Zygotic gene activation
dsRNA	Double-strand RNA
siRNA	Small interfering RNA
NICD	Notch1 intracellular domain
RBPJ	Recombination signal binding protein for immunoglobulin kappa J region
CSL	CBF1, Suppressor of Hairless, Lag-1
MAM	Mastermind
EGFP	Enhanced green fluorescent protein
hCG	Human chorionic gonadotropin
KSOM	Potassium-supplemented simplex optimized medium
hKSOM	KSOM with HEPES
