# Supplementary material for: Impaired Blastocyst Formation in Lnx2-Knockdown Mouse Embryos
Source: Int J Mol Sci. 2023 Jan 10;24(2):1385. doi: 10.3390/ijms24021385 (PMC9867088; doi:10.3390/ijms24021385)
Supplement: Supplementary file 1 [file ijms-24-01385-s001.zip › ijms-2097646-supplementary.pdf]

Supplementary Data

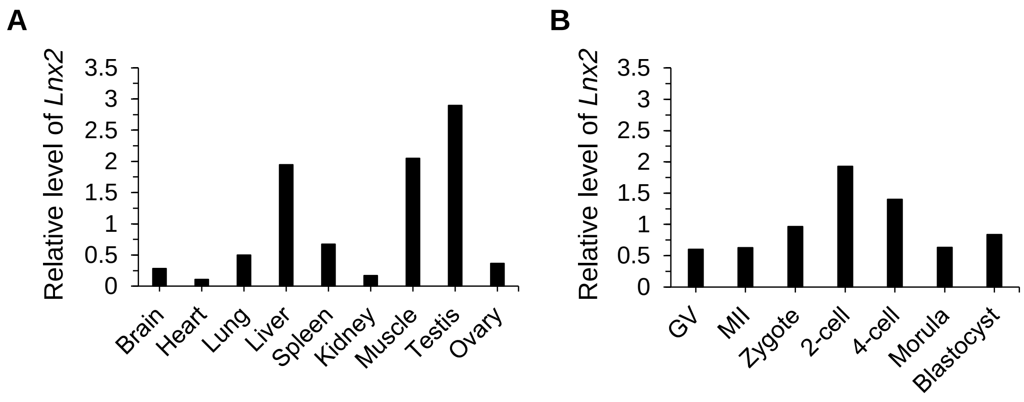

Figure S1. Quantification of the RT-PCR data shown in Figure 1. The intensities of the *Gapdh* and *Lnx2* bands in tissues (A) and preimplantation embryos (B) were measured using image J. The *Lnx2* levels were normalized the *Gapdh* values.

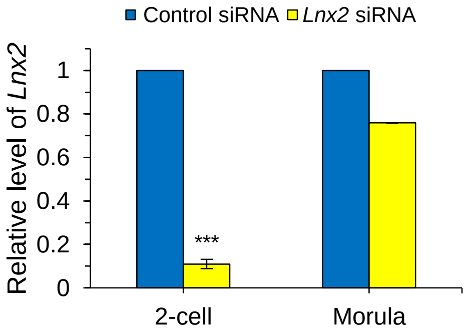

Figure S2. Zygotic injection of *Lnx2* siRNA was restored at morula stage. Zygotes (24 h post hCG injection) were injected with 20 μM of *Lnx2* siRNA mixture or control siRNA (injection control). A) cDNA was synthesized from 2-cell (48h post hCG injection) and morula (90 h post hCG injection). Values present means ± SD; ***$P < 0.001$ (experiment were repeated three times, Student's *t* test). Expression levels of *Lnx2* expression was normalized to that of *Gapdh*.

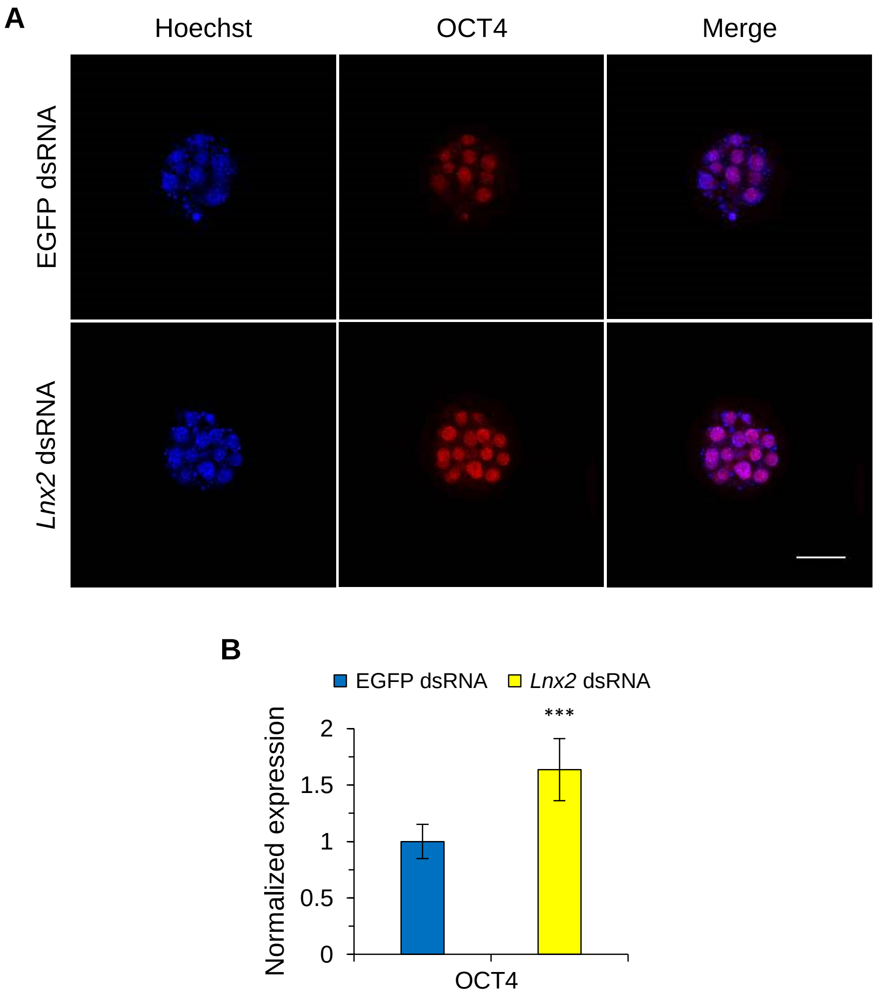

Figure S3. OCT4 expression was increased in *Lnx2* knockdown embryos. Zygotes (24 h post hCG injection) were injected with 1 mg/ml of *Lnx2* dsRNA or 1 mg/ml of EGFP dsRNA (injection control). (A) OCT4 expression in control embryos and knockdown embryos at 90 h post hCG injection. Scale bar, 40 μm. Hoechst staining was used for nuclear staining. (B) Quantification of OCT4 expression in control embryos and knockdown embryos at 90 h post hCG injection. Data are presented as mean ± SEM; ***$P < 0.001$ (expression measured in 15 control embryos and 22 knockdown embryos for OCT4, Student's *t* test).

Table S1. List of primers used for RT/qRT-PCR

Gene	Size (bp)	Forward primer	Reverse primer
Gapdh[a]	174	5'- TCCGTGTTCCTACCCCCAATG -3'	5'- GGGAGTTGCTGTTGAAGTCGC -3'
Gapdh[b]	492	5'- AGCCAAAAGGGTCATCATCTCCG -3'	5'- TCCTCAGTGTAGCCCAAGATGCC -3'
Gapdh[c]	986	5'- TGAAGGTCGGTGTGAACGGATTTGGC -3'	5'- CATGTAGGCCATGAGGTCCACCAC -3'
Lnx2[a]	173	5'- CCACACCAATCAGCCCTTCTTC -3'	5'- GCGTGACCTTGTTCCTCTGTTC -3'
Lnx2[b,c]	628	5'- AATGGATCCCTCAATTCTTCCTGCAGG -3'	5'- TTAGAATTCTACGCAACAAAGCTTTCC -3'
Oct4[a]	186	5'- AAGAACATGTGTAAGCTGCGGCCC -3'	5'- GGAGGGCTTCGGGCACTTCAGAAA -3'
Cdx2[a]	155	5'- GACTTCCTGTCCCTTCCCTCGTCT -3'	5'- CCTCCCGACTTCCCTTCACCATAC -3'
Nanog[a]	224	5'- GGGTCTGCTACTGAGATGCTCTGC -3'	5'- CTGTCCTTGAGTGCACACAGCTGG -3'
Sox2[a]	199	5'- TAGAGCTAGACTCCGGGCGATGA -3'	5'- CTCCTTCCTTGTTTGTAACGGTCC -3'
Gata6[a]	334	5'- CCTTATGGCGTAGAAATGCTGAGG -3'	5'- ATACTTGAGGTCACTGTTCTCGGG -3'
Klf2[a]	181	5'- GCTAGATGCCTTGTGAGAAATGCC -3'	5'- CTACCGTGATTCCTCCAAAGATCC -3'
Notch1[a]	201	5'- GCGAAGTGGACATTGACGAG -3'	5'- GGCATAAGCAGAGGTAGGAG -3'
Hes1[a]	148	5'- AGCCAAAAGGGTCATCATCTCCG -3'	5'- AATGCCGGGAGCTATCTTTC -3'
Hes5[a]	139	5'- AACACAGCAAAGCCTTCGCC -3'	5'- AGCAGCTTCATCTGCGTGTC -3'
Yap1[a]	109	5'- CAGGAATTATTTCGGCAGGC -3'	5'- CATCCTGCTCCAGTGTAGGC -3'
Taz[a]	175	5'- GCCGGTTCCGGGGATAAAG -3'	5'- GAAGGACTCCGGGAGGATCT -3'
Lnx1[a]	243	5'- TGGTCCACATCATCATCCAG -3'	5'- TGGTCCACATCATCATCCAG -3'

[a] : qRT-PCR, [b] : RT-PCR (Figure 1A), [c] : RT-PCR (Figure 1B)
